# Perceptual comparisons induce lasting and generalizing changes to face memory reports

**DOI:** 10.1186/s41235-024-00584-4

**Published:** 2024-09-02

**Authors:** Jerrick Teoh, Joseph M. Saito, Yvanna Yeo, Sophia Winter, Keisuke Fukuda

**Affiliations:** 1 Department of Psychology, University of Toronto Mississauga, Mississauga, Canada; 2 Department of Psychology, University of Toronto, Toronto, Canada

## Abstract

Humans are often tasked to remember new faces so that they can recognize the faces later in time. Previous studies found that memory reports for basic visual features (e.g., colors and shapes) are susceptible to systematic distortions as a result of comparison with new visual input, especially when the input is perceived as similar to the memory. The current study tested whether this similarity-induced memory bias (SIMB) would also occur with more complex face stimuli. The results showed that faces that are just perceptually encoded into visual working memory as well as retrieved from visual long-term memory are also susceptible to SIMB. Furthermore, once induced, SIMB persisted over time across cues through which the face memory was accessed for memory report. These results demonstrate the generalizability of SIMB to more complex and practically relevant stimuli, and thus, suggest potential real-world implications.

## Significance statement

Humans rely on the ability to remember faces accurately so that the faces can be reported accurately later in time (e.g., making friends in new schools and serving as an eyewitness). The current study demonstrated that, similar to memory reports of more simplistic visual information like colors and shapes, face memory reports can be systematically distorted as a consequence of comparisons with similar faces. Furthermore, this similarity-induced memory bias persisted over time and generalized across retrieval cues that were learned separately. Thus, the current results suggest caution when trusting the accuracy of face memory reports made after the face memory was compared to similar faces (e.g., after a lineup test for eyewitness testimony).

## Introduction

In everyday life, humans often have to remember faces of other individuals over a period of time so that the faces can be recognized later. To do so, visual working memory (VWM) allows active maintenance of a finite amount of visual information registered through vision or retrieved from visual long-term memory (VLTM) (e.g., Cowan, 2001; Draschkow et al., 2021; Fukuda & Woodman, 2017; Luck & Vogel, 2013) so that it can be compared with new perceptual input to guide our behavior. Theories propose that VWM enables such interaction with new perceptual input by utilizing the visual cortex for active maintenance of memory representations (Harrison & Tong, 2009; Rademaker et al., 2019; Serences et al., 2009). In support of this view, studies have consistently demonstrated reliable interactions between VWM representations and new visual input (Bae & Luck, 2019; Kang et al., 2011; Sun et al., 2017; Teng & Kravitz, 2019). Specifically, when holding simple visual information in VWM, an encounter with new visual input has been shown to bias the VWM representation and vice versa (Kang et al., 2011; Teng & Kravitz, 2019).

Although the interactions between VWM representations and new percept is essential for many goal-directed behaviors, it can pose serious problems when our goal is to maintain VWM representations as accurately as possible (e.g., maintaining accurate representation of the face of the criminal). To this end, recent studies demonstrated that a memory report of a VWM representation gets distorted after the VWM was used for perceptual comparisons with new inputs, especially when the new inputs are subjectively similar to the memory representation (Fukuda et al., 2022). More precisely, in Fukuda et al. (2022), participants are presented with a single simplistic target stimulus (i.e., a colored circle or a geometric shape) to remember over a brief delay, after which they reproduced the target as precisely as possible by selecting a stimulus from a continuous wheel. Critically, in some of the trials (i.e., comparison trials), a pair of probe stimuli were presented during the delay, and participants had to choose which probe stimulus was similar to the target stimulus maintained in their VWM. Here, the researchers found that the reproduced targets in the comparison trials were biased in the direction of the probe that was judged to be similar to the target. Importantly, this similarity-induced memory bias (SIMB) is significantly larger than the bias observed when the new inputs were perceived but ignored (Saito et al., 2022). Additionally, the SIMB accumulates or cancels its magnitude as a function of the number of similarity judgments and the direction of the similar probe (Fukuda et al., 2022; Saito et al., 2023a, 2023b). These results implicate the causal role that similarity judgments play in modulating the memory bias. Furthermore, once induced, the SIMB persisted over a 24-h delay (Saito et al., 2023a, 2023b), indicating that the SIMB incurs a lasting change to the memory representation.

Although the SIMB has been demonstrated with a variety of stimuli (e.g., color, geometric shapes and real-world objects), their similarity has been defined using a single feature space (e.g., color, shape). However, many real-world objects vary in multiple features and therefore, their similarities are defined based on multiple features (e.g., Hout et al., 2014; Valentine, 1991). Similarly, human faces are composed of multiple features (e.g., eyes, nose, mouth, skin tone) that vary across individuals, and therefore, their similarities are assessed in a multidimensional manner (Busey, 1998; Chang et al., 2017; Hopper et al., 2014; Nestor et al., 2016). Thus, to evaluate the practical importance of the SIMB, it is critical to examine whether the SIMB also applies to multidimensional and practically relevant visual stimuli like faces. Doing so in a systematic manner, however, requires the establishment of continuous stimulus space for faces. Thus, Experiments 1a and 1b were conducted to develop and empirically validate continuous face spaces (i.e., face wheels).

## Experiments 1a and 1b

To develop continuous face spaces (i.e., face wheels), four distinct artificial faces were generated for each of the eight ethnicity (African, East Asian, European, and South Asian) x gender (female and male) groups to serve as seed faces for the corresponding face wheels. The four seed faces were then systematically morphed to construct a circular continuous space for each ethnicity x gender group (see Method for more details). To empirically validate their circular continuity, face similarity judgment experiments (Experiments 1a and 1b) were conducted. In these experiments, participants were first presented with a face triplet drawn from the same face wheel (e.g., African male), and they were asked to judge whether the face on the left or right was more similar to the center face. Subsequently, the left and center faces remained on the computer screen, and participants rated how similar the left face was to the center face. Lastly, the left face was replaced with the right face, and participants rated the similarity between the two. The similarity ratings produced for the face pairs were then submitted to multidimensional scaling (MDS) analysis to empirically confirm the continuity of the face wheels (Hout et al., 2014; Kriegeskorte & Mur, 2012; Li et al., 2019). To preview the results, the MDS analysis demonstrated that the 2-dimensional similarity space for each face wheel approximated circularity, providing support for the circular continuity of each face wheel.

### Method

#### Participants

A total of 183 (136 females, 46 males, 1 no response; 90 and 93 participants for Experiments 1a and 1b, respectively) Psychology students at the University of Toronto Mississauga participated in our experiments to fulfil a course requirement for an introductory psychology course. Prior to the experiments, all participants signed a consent form approved by the Research Ethics Board at the University of Toronto.

#### Stimuli and apparatus

Eight (4 ethnicities: African, European, East Asian, and South Asian) × 2 genders (Female and Male) continuous face wheels were created using artificial faces generated by Facegen Modeller (Singular Inversions Inc., 2009). More specifically, for each ethnicity and gender (e.g., African, female), four faces were manually designed as seed faces. The four seed faces were placed at 0, 90, 180, and 270 degrees on a circular space. Then, two consecutive seed faces were morphed to create 89 faces that would be placed between the two seed faces in an equidistant manner (i.e., every 1°). For example, 15° face was created by morphing 0° and 90° with 5 to 1 ratio. 30° face was created by morphing 0° and 90° with 2 to 1 ratio. The resultant continuous face space was called a “face wheel”, and a separate face wheel was generated for each ethnicity and gender combination, thus resulting in eight face wheels in total. All the stimuli are publicly posted at the OSF website (https://osf.io/r6xne/).

#### Procedures

**Fig. 1:**
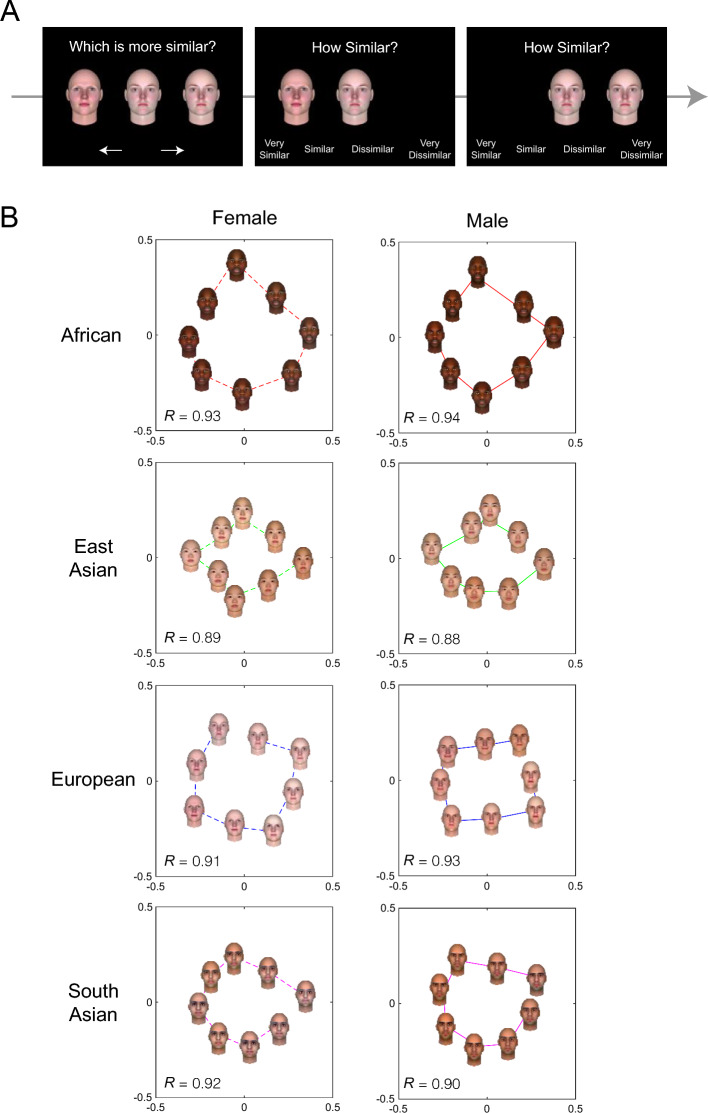
A Schematic and Results of Face Wheel Validation (Experiments 1a and 1b). Panel **A** shows the schematic of Face Wheel Validation Experiment (Experiment 1a). Panel **B** shows the best-fit 2-D similarity space for each face wheel

In Experiments 1a and 1b, each trial started with a presentation of a central fixation cross. 500 ms later, three faces (each face occupied 12% of the computer screen) drawn from the same face wheel were presented on the computer screen along with two arrows (i.e., left and right arrows; Fig. 1A), and participants were asked to indicate whether the left or right face was more similar to the center face by clicking on the corresponding arrow. The face triplets presented were chosen from 0°, 45°, 90°, 135°, 180°, 225°, 270°, 315° from a seed face without replacement. Upon response, the left and center faces remained on the screen along with a Likert scale, and participants judged how similar the left face was to the center face by choosing one the options on the Likert scale. In Experiment 1a, a four-point Likert scale (*Very similar*, *Similar*, *Dissimilar*, and *Very dissimilar*) was used, whereas in Experiment 1b, a six-point Likert scale (*Very similar*, *Moderately similar*, *A little similar*, *A little dissimilar*, *Moderately dissimilar*, and *Very dissimilar*) was used. Different response scales were used to ensure that the result was not limited to a specific response scale. Upon response, the left face disappeared as the right face appeared, and participants judged how similar the right face was to the center face with the same procedure. After providing the response, the computer screen turned blank, and participants hit the spacebar to initiate the next trial. Participants performed four blocks of 60 trials, thus submitting 60 pairwise similarity ratings of faces for each face wheel in a randomized order.

#### Analysis

To allow a combination of datasets from Experiment 1a and 1b, the pairwise similarity ratings was first standardized within each individual and a multidimensional scaling analysis using a MATLAB function (mdscale.m with a max iteration of 5000) was conducted to create a two-dimensional representation of the standardized similarity space for each face wheel. To estimate the circular continuity of the face wheels, the mean perceived similarity score for each face pair (8 center faces × 7 lateral faces = 56 pairs of faces) was correlated with the estimated Euclidian distance between the assumed positions of the corresponding faces on a circle with a radius of 1. For example, the estimated Euclidian distance between two faces that are 180 degrees apart is 2, and the estimated distance for the two faces that are 90 degrees apart is a square root of 2.

### Results

Figure 1B shows the two-dimensional representation of the face spaces for each face type along with four seed and four intermediate faces used in Experiments 1a and 1b. As can be seen, each intermediate face was placed closest to the corresponding seed faces that were morphed to create the intermediate faces, thus demonstrating the continuity of face wheels. To further validate the circular continuity, the similarity ratings for each pair was correlated with the Euclidian distance expected if the two-dimensional space was indeed circular. Here, the correlations were highly significant, explaining > 77% of the variance (*r*(54) > 0.88, *p* < 0.01) for each face wheel. These results empirically support the circular continuity of our face wheels.

### Discussion

Experiments 1a and 1b provided support for the circular continuity of the eight face wheels. Thus, Experiment 2 utilized these face wheels to test whether the SIMB occurred for multidimensional face stimuli.

## Experiment 2

With the empirically validated continuous face wheels, Experiment 2 investigated whether the SIMB would occur for multidimensional face stimuli when they were perceptually encoded into VWM. To do so, participants were presented with one target face from a face wheel to remember over a brief retention interval. Critically, in some trials, a pair of faces (i.e., probe faces) from the same face wheel was presented during the retention interval, and participants indicated which of the pair was more similar to the target face. After the retention interval, participants reproduced the target face as accurately as possible using the face wheel. If the SIMB also occurs for face memories, then the memory recall for the target face should be systematically biased in the direction of the probe face judged to be similar.

### Method

#### Participants

Previous studies that utilized more simplistic stimuli (e.g., colour, shape) reported large effect sizes (Cohen’s* d* > 0.8*)* (Fukuda et al., 2022; Saito et al., 2022)*.* However, considering the complexity of the current stimuli, a half-sized effect (Cohen’s d = 0.4) was anticipated. As a result, the target sample size was set as at least 68 participants to establish a statistical power of 0.9 (*p* < 0.05, two-tailed).

Participants were recruited on a weekly basis in return for course credits for Psychology courses until the number of qualified participants reached our target sample size. All participants reported normal hearing and normal or corrected-to-normal vision. For Experiment 2, 73 University of Toronto Mississauga (UTM) students (mean age = 19.0 years, *SD* = 2.3 years, 19% Male, 81% Female) participated. Data from four participants were excluded from the analyses because they failed to complete 50% or more of the similarity judgments within the pre-determined time window (2000 ms, see Procedures). As a result, data from 69 participants were subjected to the analyses.

#### Stimuli and apparatus

**Fig. 2:**
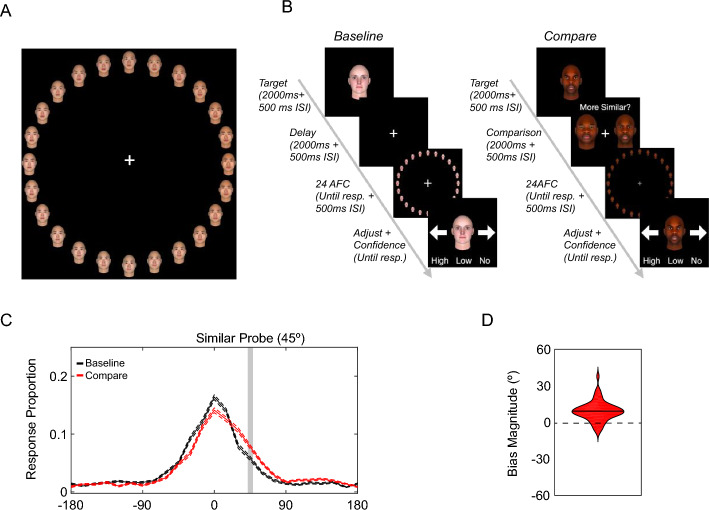
An example face wheel and the schematic and the results of Experiment 2. Panel **A** shows an example face wheel for East Asian male faces. Panel **B** shows the schematics for the baseline and compare conditions in Experiment 2. Panel **C** shows the distribution of signed response offsets for Experiment 2. The dotted lines represent the within-subject standard error of the means. Panel **D** shows the violin plot for the bias magnitude. The black solid line indicates the mean bias magnitude

Stimuli were sampled from the eight (4 ethnicities (African, European, East Asian, and South Asian) × 2 genders (Female and Male)) continuous face wheels validated in Experiments 1a and 1b (see Fig. 2A for the Asian male face wheel as an example).

Experiment 2 was conducted online using Inquisit Player 6 (millisecond, 2020) downloaded and installed locally on each participant’s computer. Experimenters provided instruction and monitored participants’ progress throughout the experiment using Zoom conferencing (Zoom Video Communications, 2020).

#### Procedures

After providing informed consent for the protocol approved by the University of Toronto Research Ethics Board, participants performed a face working memory task (Fig. 2B). Each trial began with a presentation of a fixation cross at the centre of the screen. 500 ms later, a target face (occupying 12% of the screen) was presented at the centre of the screen for 2000 ms, and participants were instructed to remember the face as precisely as possible. The target face was sampled pseudo-randomly from the four seed faces of eight face wheels (32 seed faces in total) so that each seed face was presented four times throughout the experiment. In two of the four trials for each seed face, the target face presentation was followed by a 3000 ms maintenance interval (*Baseline* trials), after which a face wheel was presented to prompt the reproduction of the target face. The face wheel was composed of the target face and 23 faces (each occupying 0.48% of the screen) sampled equidistantly (15° apart) from the corresponding race and gender group (radius = 40% of the height of the screen). Participants reported the target face by clicking on one of the face on the face wheel. Importantly, the face wheel was randomly rotated on a trial-by-trial basis to minimize the systematic effect of potential response strategies (e.g., avoiding cardinal directions). After selection, the clicked face was displayed at the centre of the screen with left and right arrows on the corresponding sides. Participants then used their mouse to click on the arrows to make fine adjustments to their selection to obtain the exact face they remembered. Clicking the left arrow changed the center face by 15° in a clockwise direction on the face wheel, and clicking the right arrow did so in a counter-clockwise direction. Once the center face matched with the exact face the participants remembered, they indicated their confidence in the accuracy of their memory report by clicking on one of the three buttons (i.e., ‘High’ for high confidence, ‘Low’ for low confidence, ‘No’ for no confidence). The accuracy of the memory report was emphasized, and therefore, no time limit was imposed.

In the remaining two trials for each seed face (*Compare* trials), participants completed one similarity judgment during the maintenance interval. 500 ms after the offset of the target face, two face probes (each occupying 12% of the screen) sampled from the same face wheel as the target face were presented on either side of the screen. One of the face probes was 45° away from the target face (similar probe), while the other face probe was 180° away from the target face (dissimilar probe). In one of two similarity judgment trials for each seed face, the similar probe was sampled from the clockwise direction, and it was sampled from the counter-clockwise direction in the other trial. Participants’ task was to select which of the face probes was similar to the target face within 2000 ms. A failure to do so rejected the trial from the analyses. The face probes remained on the computer screen for 2000 ms irrespective of the response time for the similarity judgment, after which the computer screen remained blank for 500 ms. Subsequently, participants reported the target face with the same procedure as the baseline trials. In total, participants performed four blocks of 32 trials (16 *Baseline* and 16 *Compare* trials) in a randomized order.

#### Analysis

To measure the SIMB, the signed response offset for each trial was computed using the following procedure. First, the angular difference was computed between the memory report and the target face on the face wheel. The direction of the signed response offset was then determined so that the positive offset indicated response offset towards the similar face presented in the similarity judgment. For the baseline condition, the direction of the response offset was randomly assigned because no similar face was presented in the baseline condition. The signed response offsets were then averaged across all trials for each condition. Furthermore, to ensure that the SIMB also occurs when participants indicate high subjective confidence in the accuracy of memory reports, we repeated the same analyses for high confidence reports only.

### Results

#### Face working memory report is distorted by a perceptual comparison toward a similar input

As can be seen in Fig. 2C, the response offsets for the baseline condition clustered tightly around 0°, suggesting that participants maintained an accurate representation of a target face in their VWM. This was further collaborated by their near-ceiling accuracy for the perceptual comparison task (mean Accuracy = 0.95%, S.D. = 0.053%). More importantly, the distribution of the signed response offsets was shifted in the direction of the similar probe. Consistent with this observation, the mean bias magnitude for the compare condition was significantly higher than zero (Fig. 2D, t(68) = 8.20,* p* < 0.001, *Cohen’s d* = 0.99, *95% CI* [6.67° 10.96°] for all trials; *t*(68) = 6.17, *p* < 0.001, *Cohen’s d* = 0.74, *95% CI* [5.29° 10.34°] for high-confidence trials only), indicating that a report for a face VWM representation was biased by a perceptual comparison in the direction of a similar face.

### Discussion

Experiment 2 demonstrated a robust SIMB effect for face VWM when faces are perceptually encoded into VWM. Similarly to the previous demonstration of SIMB with simplistic stimuli (e.g., color, shapes, Fukuda et al., 2022; Saito et al., 2022), the SIMB was observed even when participants indicated high confidence in the accuracy of the memory reports. This demonstrates that the SIMB is not a strategic bias that participants engage in when they are not sure of their memory accuracy. Taken together, the results indicate that the SIMB is not limited to simplistic stimuli whose similarity is defined along a single feature space (e.g., color) but also applies to more practically relevant multidimensional stimuli.

## Experiment 3

Experiment 3 examined the vulnerability of face visual long-term memories (VLTMs) to SIMB when retrieved into VWM. To do so, participants first encoded eight faces (one face per face wheel) along with two attributes (i.e., name and subject of study) into their VLTM (the learning phase). After learning was complete, participants were presented with one of the attributes and tasked to retrieve the associated target face from VLTM into their VWM (the retrieval practice phase). Upon retrieval, a pair of probe faces was presented, and participants judged which of the probe faces was more similar to the retrieved target face. Subsequently, participants recalled the target face on the face wheel. If a face VLTM is also vulnerable to the SIMB, then the memory report in the retrieval practice phase should be biased in the direction of the probe face judged to be similar.

After the retrieval practice phase, participants were again presented with one of the attributes and recalled the associated face using the face wheel (the final recall phase). If the SIMB induced during the retrieval practice phase persisted over time, then the memory report in the final recall phase should also be biased in the direction of the similar probe face presented earlier in the retrieval practice phase. Furthermore, if the SIMB is specific to the retrieval cues used to access the target face memory (e.g., Godden & Baddeley, 1975; Hupbach et al., 2008; Tulving & Thomson, 1973), the SIMB should only be observed when the final recall was triggered with the cues used during the retrieval practice phase. On the other hand, if the SIMB generalizes to other retrieval cues, then the SIMB should also occur when the final recall is prompted with the cue not used during the retrieval practice phase.

### Method

#### Participants

Experiment 2 demonstrated large effects (Cohen’s d > 0.743) for all the comparisons of interest. However, because of the reduced number of trials per participant in Experiment 3, a medium effect size (Cohen’s *d* = 0.5) was anticipated. Thus, the target sample size was set as at least 44 participants to achieve the statistical power of 0.9 (*p* < 0.05, two-tailed). Data collection was continued on a weekly basis until the number of qualified participants reached our target sample size. As a result, 56 UTM students (mean age = 19.5 years, *SD* = 2.8 years, 30% Male, 70% Female) participated in return for course credits for Psychology courses. Data from three participants were removed because they failed to achieve 75% accuracy on the similarity judgments in the retrieval practice. As a result, data from 53 participants were subjected to the analyses.

### Procedures

#### Face working memory task

**Fig. 3:**
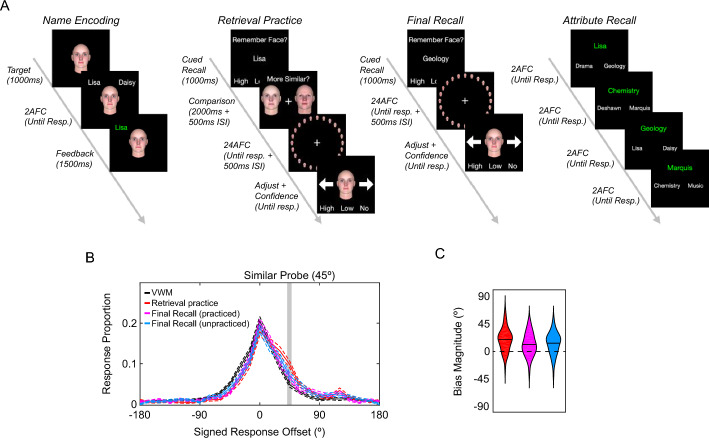
Results of Experiment 3. Panel **A** shows the schematics for the name encoding task, retrieval practice task, final recall task, and attribute recall task. Panel **B** shows the distribution of signed response offsets for Experiment 3. The dotted lines represent the within-subject standard error of the means. Panel **C** shows the violin plots for the bias magnitudes. The black line indicates the mean bias magnitude for each condition

Following the same informed consent procedure as in Experiment 2, participants first performed two blocks (32 trials each) of face working memory task (See Fig. 3A for detailed schematics). Each trial began with a target face presented at the centre of the screen for 2000 ms, and participants were instructed to remember the face as precisely as possible. The target face was pseudo-randomly sampled from four seed faces in the eight face wheels so that each seed face was presented in four trials. The target face presentation was followed by a 1000 ms maintenance interval. The target face was then reported using the same procedure as Experiment 2.

#### Profile encoding task

Next, participants learned the names and majors of eight target faces by performing the name and major encoding tasks. Each target face was one seed face randomly chosen from each of the eight face wheels. In the name encoding task, one target face was presented with two names (Fig. 3A), and participants clicked on the correct name associated with the face. Upon selection, the correct name turned green for 1500 ms. The major encoding task was identical to the name encoding task except that the target face appeared with two majors. Participants performed eight blocks of each encoding task in a pseudo-random order. In each block, each target face was presented twice in a pseudo-random order. As a result, participants completed 128 trials in total.

#### Retrieval practice task

Upon completion of the encoding tasks, participants performed the retrieval practice task. In this task, participants were first presented with one of the cues associated with a target face (i.e., name or major, Fig. 3A) at the centre of the screen. Here, the participants’ task was to retrieve the associated target face and report their confidence in the accuracy of the retrieved face memory by clicking on one of the ‘High’, ‘Low’, and ‘No’ buttons. Following the confidence report, participants were presented with a pair of a similar and a dissimilar face (45° and 180° away from the target face, respectively) to the target face, and they were asked to click on the similar face. After the similarity judgment, participants reported the target face using the same procedure as Experiment 2.

Importantly, for each target face, the cue used to trigger the retrieval (e.g., either name or major) and the similar and dissimilar faces in the similarity judgments remained the same across all retrieval practice trials (two trials for each target face) for the specific target face for each participant. This ensured that one of the cues for each target face remained unused to trigger the retrieval of the target face, and the direction of the SIMB induced for each target face was consistent across all the retrieval practice trials. Equally importantly, the assignment of the retrieval cue and similar and dissimilar faces for each target face was randomized across participants. In total, participants completed 16 trials (2 trials for each of eight target faces) in a randomized order.

#### Final recall task

Subsequently, participants completed the final recall task. This task was identical to the retrieval practice task except for two details (Fig. 3A). First, there were no similarity judgments. Second, both practiced and unpracticed cues were used in separate trials (two trials each) to trigger the memory retrieval. Each target face was recalled twice using the practiced cue and twice using the unpracticed cue in a pseudo-random order. In total, participants completed a one block of 32 trials in a randomized order.

#### Attribute recall task

Finally, participants completed an attribute retrieval task to verify that they successfully maintained the association between the face and the two cues. In this task, one cue from one of the attributes (e.g., Lisa from the name attribute, Fig. 3A) was displayed in the centre of the screen in green font, along with two cue alternatives from the other attribute (e.g., Drama and Geology from the major attribute, Fig. 3A) in white. Participants were instructed to click on one of the white alternatives that was associated with the green cue. Each of the cues in eight learnt cue pairs served as the green cue in a pseudorandom order. Thus, participants completed 16 trials in one block.

### Results

#### Face long-term memory report is distorted by a perceptual comparison toward a similar face

First, to ensure that participants successfully formed an association for each face with corresponding cues (i.e., name and major), encoding performance was examined. The accuracy of the encoding task monotonically increased throughout the blocks and reached a ceiling (mean accuracy: 0.49, 0.80, 0.93, 0.97, 0.97, 0.97, 0.99 and 0.99, for blocks 1–8, respectively for the name task; mean accuracy: 0.55, 0.88, 0.95, 0.97, 0.97, 0.97, 0.99 and 0.99, for blocks 1–8, respectively for the major task), suggesting that participants successfully formed the association prior to the retrieval practice task. Furthermore, the ceiling-level comparison accuracy (mean accuracy: 0.95) during the retrieval practice task and the attribute recall accuracy (mean accuracy: 1.00) confirm that participants successfully maintained the associated cues and used them to retrieve face memories.

As can be seen in Fig. 3B, the signed response offset distribution for the retrieval practice condition (red line) was shifted in the direction of the similar probe. Corroborating this observation, the mean bias magnitude was significantly higher than zero (red violins in Fig. 2d, t(52) = 7.46, *p* < 0.001, *Cohen’s d* = 1.02, *95% CI* [13.86° 24.07°] for all trials; *t*(52) = 7.44,* p* < 0.001, *Cohen’s d* = 1.02, *95% CI* [14.23° 24.74°] for high confidence trials only), indicating that a report for a face VWM representation retrieved via an associative cue was biased by a perceptual comparison in the direction of a similar face.

#### The SIMB for face memories generalizes across time and retrieval cues

Lastly, the persistence and cue generalizability of the SIMB was examined. As can be seen in Fig. 3C, the signed response offset distributions for the practiced cue condition in the final recall task (magenta line) were shifted in the direction of the similar probe. Corroborating this observation, the mean bias magnitude was significantly higher than zero (magenta violin in Fig. 2d, t(52) = 4.36, *p* < 0.001, *Cohen’s d* = 0.60, *95% CI* [6.02° 16.29°] for all trials; *t*(52) = 4.93, *p* < 0.001, *Cohen’s d* = 0.68, *95% CI* [7.56° 18.02°] for high confidence trials only), although the bias magnitude was smaller when compared to that observed during the retrieval practice task (*t*(52) = 2.57, *p* < 0.05, *Cohen’s d* = 0.35 for all trials; *t*(52) = 2.12, *p* < 0.05, *Cohen’s d* = 0.29 for high confidence trials only). These results indicate that the similarity-induced memory bias (SIMB) persisted across time, though reduced, when the face memory was accessed again using the same retrieval cue as the retrieval practice task.

Similarly, the signed response offset distributions for the unpracticed cue condition in the final recall task (cyan line) were also shifted in the direction of the similar probe. Corroborating this observation, the mean bias magnitude was significantly higher than zero (cyan violin in Fig. 2d, t(53) = 5.57, *p* < 0.001, *Cohen’s d* = 0.76, *95% CI* [8.61° 18.31°] for all trials; *t*(53) = 5.42, *p* < 0.001, *Cohen’s d* = 0.74, *95% CI* [8.54° 18.59°] for high confidence trials only), although the bias magnitude was smaller when compared to that observed during the retrieval practice task (*t*(52) = 2.11, *p* < 0.05, *Cohen’s d* = 0.29; *t*(52) = 2.54, *p* < 0.05, *Cohen’s d* = 0.35 for high confidence trials only). Most critically, the SIMB magnitude was not reliably different between the practiced cue and unpracticed cue conditions (*t*(52) = 0.93, *p* = 0.36, *Cohen’s d* = 0.13 for all trials; *t*(52) = 0.28, *p* = 0.78, *Cohen’s d* = 0.04 for high confidence trials only). Taken together, these results demonstrate that the SIMB generalized across retrieval cues that were learned separately.

### Discussion

Experiment 3 demonstrated that, once introduced, the SIMB for the face memories persisted over time although its amplitude reduced over time. The time-based reduction of the SIMB was not necessarily predicted, but one possible explanation is that the face generated to report the target face during the retrieval practice task could have also biased the memory report in the final recall task. Since the face generated for the memory report at the retrieval practice was generally closer to the target than to the probe, this face could have biased the memory report closer to the target. Future studies should examine this possibility directly. More critically, the magnitude of the persisted SIMB was the statistically indistinguishable when the face memory was accessed using the practiced and unpracticed retrieval cues. This result revealed a generalizability of the SIMB across retrieval cues.

## General discussion

The current study examined whether the SIMB would occur for a practically relevant and multidimensional stimuli, namely, faces. To do so, Experiments 1a and 1b first constructed and empirically validated eight continuous face wheels. Next, using the face wheels, subsequent experiments demonstrated that SIMB occurred for face memories when they were perceptually encoded in to VWM (Experiment 2) and when they were retrieved from VLTM (Experiment 3). Although a past study demonstrated that face memory reports can be distorted by a presentation of distractor faces (Mallett et al., 2020), the current study is the first to report that face memory reports can be systematically biased through a goal-driven usage of the face memories. Additionally, the results suggest that retrieving a VLTM representation into VWM restores the VLTM into a malleable state and allows the memory to interact with new perceptual inputs. Furthermore, this interaction resulted in lasting memory distortions (cf., memory reconsolidation; Hupbach et al., 2007, 2009) where the memory distortions persisted in subsequent memory retrievals even when they were triggered by a different associative retrieval cue that was learned separately. To our knowledge, the current study is the first to demonstrate the cue generalizability of the SIMB. These findings demonstrate the generalizability and robustness of the SIMB that are fundamental in explaining a wide variety and ubiquity of memory distortions observed not only in the lab settings but in real-world scenarios (e.g., eyewitness testimonies).

### Representational shift or probabilistic confusions?

The current findings are not without limitations. Although the results demonstrated a robust shift in memory reports, it does not necessarily indicate a verbatim shift of the original memory representation because probabilistic confusion between the original memory representation and its similar comparator can also produce a similar shift in memory reports (Fukuda et al., 2022). Indeed, a recent work applied computational modeling on continuous memory report data and demonstrated both mechanisms can underlie systematic shifts in memory reports with more simplistic stimuli (e.g., color and shape; Saito et al., 2023a, 2023b). More precisely, when participants perceive subjective similarity between the original memory representation and the new perceptual input, the shift in memory reports is better explained by the shift of the original memory representation. However, when participants perceive “sameness” between them, the shift in memory reports is better accounted for by the probabilistic confusion between the two. Since the current data do not allow the adoption of the same approach due to mechanical constraints (e.g., limited number of trials and less granularity in memory responses), future studies should examine whether the SIMB reported with multi-dimensional stimuli are better explained by representational shift or probabilistic confusion. Considering that both memory bias and memory replacement can have practical implications (e.g., inaccurate eyewitness testimony), such future work will also be meaningful in applied contexts.

### Dichotomy versus continuity of perceived similarity judgments

In the current study, participants were tasked to judge the similarity between a face memory and probe faces in a dichotomous manner (i.e., similar or dissimilar). Although such dichotomous judgments are ubiquitous in real life, face similarity is likely estimated continuously rather than dichotomously. In fact, this continuous perceived similarity is what allowed the construction of the face wheels in Experiments 1a and 1b. This notion, then, naturally motivates an interesting hypothesis that the magnitude of the SIMB may scale continuously with the extent of perceived similarity. Unfortunately, the mechanistic constraints of the current study (i.e., the limited number of validated face wheels and face-attribute associations individuals could reliably remember in a short period of time) prevented the characterization of the SIMB in such a continuous fashion. However, previous work using different sets of stimuli demonstrated that the SIMB occurs across a range of physical distances between the target and the probe so long as participants perceive the probe to be subjectively similar to the target (Fukuda et al., 2022; Saito et al., 2023a, 2023b; Saito et al., 2023a, 2023b). Based on these findings, a reasonable speculation would be that the magnitude of the SIMB increases as a function of the perceived similarity between the face memory and a probe face until they are so similar that they are perceived as indistinguishable (i.e., “same” instead of “similar”), at which point, the probe face will replace the original memory representation (Saito et al., 2023a, 2023b). Thus, future studies are encouraged to investigate this hypothesis directly.

Relatedly, research suggests that the face memory becomes less accurate as multiple reports are made based on it (e.g., Wixted et al., 2015, 2021). Thus, it is practically important to examine how the magnitudes of SIMB for face memories change as a function of the number of comparisons made. Although the current study is not designed to address this question due to a limited and fixed number of comparisons made for each target face memory (i.e., twice for each of eight target faces), past studies using different sets of stimuli (e.g., color and shapes) demonstrated that the SIMB magnitudes scaled with the number of comparisons made (Fukuda et al., 2022; Saito et al., 2023a, 2023b). These results allow speculation that a similar accumulation of memory bias across comparisons might also occur for face memories. Future studies should directly examine this possibility as such work will have direct implications in applied settings in which preservation of memory accuracy across multiple assessments is of the most importance (e.g., crime investigations).

### SIMB in other sensory modalities?

Although the SIMB has been demonstrated across a wide range of visual stimuli, working memory can represent information across all sensory modalities (e.g., auditory and tactile sensations) to guide behaviours. Thus, future studies should examine whether the SIMB occurs with WM representations for other sensory modalities. Given that everyday memories are multi-dimensional in nature, such studies would be critical in providing a unified account of episodic memory distortion and characterizing and potentially remedying its real-world implications (e.g., misidentification of suspects based on distorted memory reports).

## Data Availability

The data, stimuli, and codes will be publicly available at Open Science Framework (https://osf.io/r6xne/) upon publication.
